# Supplementary material for: Causes and consequences of sex-chromosome turnovers in Diptera
Source: Evol Lett. 2026 Mar 12;10(3):253–68. doi: 10.1093/evlett/qrag003 (PMC13231274; doi:10.1093/evlett/qrag003)
Supplement: qrag003_Supplemental_Files [file qrag003_supplemental_files.zip › Supplementary_Datasets.zip!/Supplementary_Datasets/Dataset_2_Graphical_representation_outgroups/Drosophila_sulfurigaster_albostrigata_chromosomes.pdf]

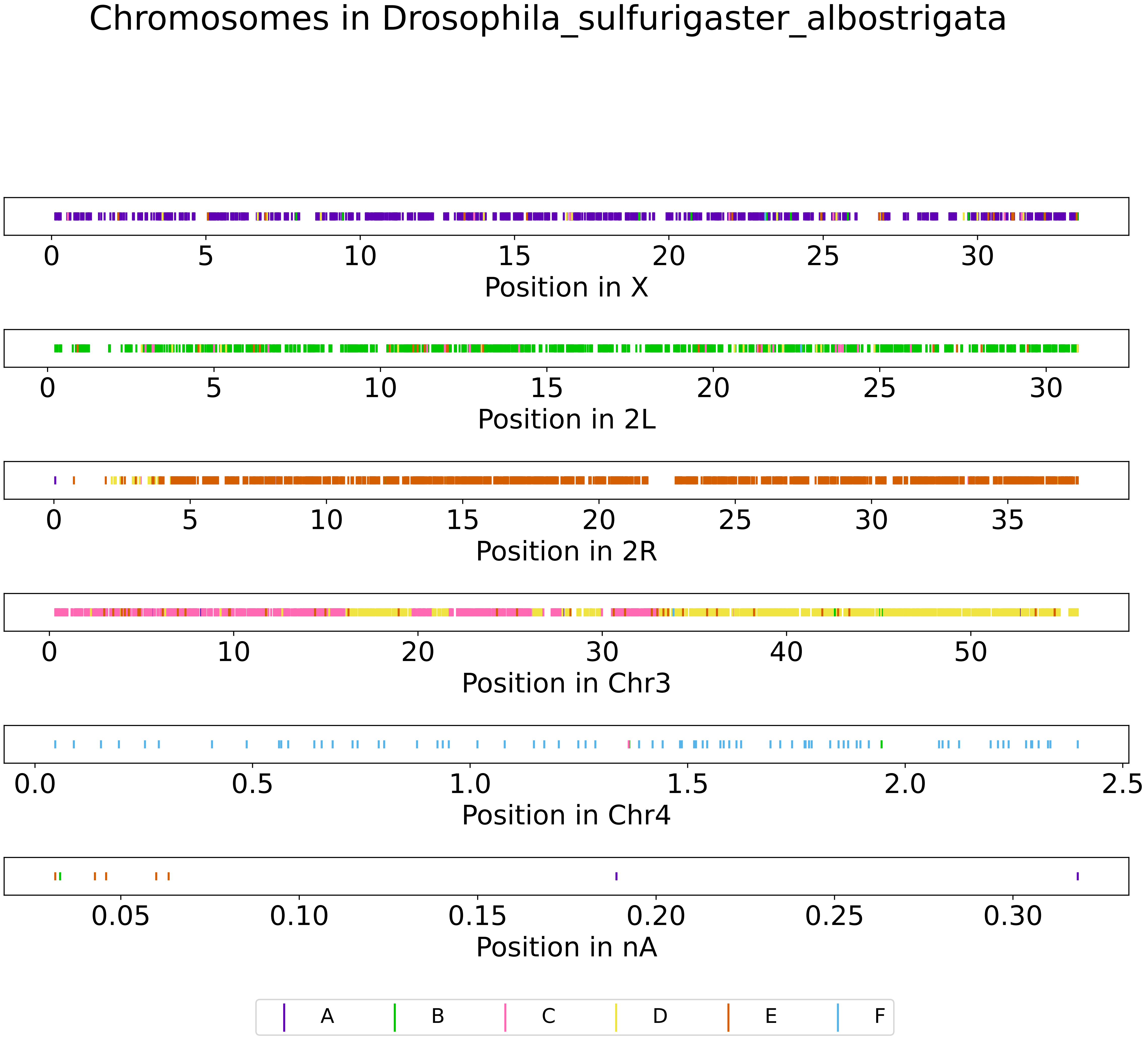
Chromosomes in Drosophila_sulfurigaster_albostrigata
0 5 10 15 20 25 30
Position in X
0 5 10 15 20 25 30
Position in 2L
0 5 10 15 20 25 30 35
Position in 2R
0 10 20 30 40 50
Position in Chr3
0.0 0.5 1.0 1.5 2.0 2.5
Position in Chr4
0.05 0.10 0.15 0.20 0.25 0.30
Position in nA
A
B
C
D
E
F
